# Genotoxicity Assessment of Three Nutraceuticals Containing Natural Antioxidants Extracted from Agri-Food Waste Biomasses

**DOI:** 10.3390/foods9101461

**Published:** 2020-10-14

**Authors:** Nadia Badolati, Raffaello Masselli, Maria Maisto, Alessandro Di Minno, Gian Carlo Tenore, Mariano Stornaiuolo, Ettore Novellino

**Affiliations:** Department of Pharmacy, University of Naples Federico II, Via Montesano 49, 80131 Naples, Italy; nadia.badolati@unina.it (N.B.); raffaello.masselli@unina.it (R.M.); maria.maisto@unina.it (M.M.); alessandro.diminno@unina.it (A.D.M.); giancarlo.tenore@unina.it (G.C.T.); ettore.novellino@unina.it (E.N.)

**Keywords:** food waste, nutraceuticals, grape pomace, apple extract, polyphenols, resveratrol, Ames test, antioxidants, Taurisolo, AnnurComplex

## Abstract

Grapes and apples are the most cultivated fruits in the Mediterranean basin and their agricultural processing is responsible for the production of a large amount of bio-waste. The reuse of this food biomass would increase the volume of recyclable/renewable biomaterial and lower the environmental impact due to the increasing demand for these biological products. To this purpose, agri-food waste from grape and apple processing have become an important source of phytochemicals, and many pharmaceutical industries are using it as starting material to produce dietary supplements, functional foods, and food additives for human consumption. In virtue of the chemical diversity and complexity of agri-food biowaste, developers and producers of nutraceuticals are advised to assess the safety of their final nutraceutical products, in compliance with European Food Safety Authority regulation. Here, we use the Ames test to assess the mutagenicity of three nutraceuticals obtained from agri-food waste biomasses: Taurisolo^®^ from grape pomace of *Vitis vinifera* L. cv ‘Aglianico’, AnnurComplex^®^ from *Malus pumila* M. cv ‘Annurca’ and Limoncella Apple Extract from *Malus domestica* B. cv ‘Limoncella’. The results showed that all three nutraceuticals were non-mutagenic.

## 1. Introduction

As a consequence of its consumeristic habit, modern society deals with an excessive demand for food, food products, and material of biological origin. The environmental impact caused by the handling of this massive request must be minimized [1]. Taking into account the increase in the world population expected in the near future (9.5 billion people in 2050 and 11.2 billion people in 2100) [2], the World Health Organization considers the achievement of this minimization a mandatory task for modern society. One of the ways environmental impact can be reduced is by enhancing renewable resources, especially those taking part in the production and processing of agricultural biomasses [3].

As described by Xia et al. [4], food waste reaches, globally, a billion tons per year, an amount that can be definitely decreased but that will be never completely erased. During food processing, ~75% in weight of the starting biological material becomes waste, contributing 140 billion tons of biomass from the food sectors generated each year around the world. A considerable part of this biomass is made up of agricultural waste (leaves, roots, stalks, bark, bagasse, straw residues, seeds, and woods). Another significant part of the agri-food waste is made by food products (mostly 2nd-best fruits and vegetables), whose weight, shape and/or aesthetic features do not reflect those requested by the modern global market.

Grapes and apples are the most cultivated fruits in the Mediterranean basin and their agricultural processing is responsible for the production of a large amount of bio-waste [5]. The reuse of food waste from grapes and apples would minimize the volume of non-recyclable/renewable material in Europe [6].

In the last decades, the pharmaceutical industry has become aware that the bio-waste originating from grapes and apples processing can be an important source of phytochemicals to be destined to cosmetic, pharmaceutical and food industries [1,6,7,8,9]. As a consequence, bio-waste has being recently used for the production of dietary supplements, functional foods, food additives and nutraceuticals for human consumption [4,9,10,11,12,13,14,15].

Europe has an area of 3.7 million he covered in vineyards and produces 177.6 million hl of wine [16,17]. Italy is among the Mediterranean countries mostly contributing to this production, with 0.7 million he of vineyards and 51 million hl of wine produced [16]. This production generates a huge amount of waste that is mainly made up of lees, skin, seeds and stalks and which management is ecologically and economically demanding [9]. This biomass, indeed, cannot be used as fertilizer because of its high content in phenolic acids that inhibits seed germination and must, thus, be burned or composted [5]. An important residue of the wine-making process is the solid waste left over after the pressing and fermentation of the grapes: the grape pomace. It can be estimated that, since 20–25% in weight of processed grapes remains as pomace, 10.5–13.1 million tons of grape pomace are produced every year in the world [1,6,8]. This residue has attracted considerable attention during the last decade by virtue of its high content of health-promoting molecules [6,18] such as stilbenes (trans-resveratrol and ε-viniferin), phenolic acids (benzoic and hydroxycinnamic acid), simple flavonoids (catechins, flavanols, and anthocyanins), tannins and oligomeric procyanidins.

Apples are very popular and frequently consumed fruits in Europe. Their processing results in 25% of the biomass being lost in the form of waste. Moreover, the 2nd-best fruits are discarded when their shape and color do not fulfill those requested for sale. Apple waste disposal is very expensive since these fruits easily ferment causing environmental pollution. Like grape pomace, apples and apple-waste contain numerous phytochemicals like dietary fibers (mainly pectin) and natural antioxidants (catechins, quercetin, procyanidins, phlorizin, rutin) and could be used as starting material to produce nutraceuticals [19,20].

Nutraceuticals containing apple extract or grape pomace extracts are becoming popular over-the-counter products by virtue of their antioxidant activity and their availability in pharmacies, supermarkets and online specialized shops [21]. They are labelled as “natural”, a term that let them be considered as “generally safe”. Indeed, the database of botanicals, compiled (and constantly updated) by European Food Safety Authority (EFSA), mentions bio-products of apple and grape processing among the natural products that can be included in nutraceutical, food supplements and functional foods in Europe [22]. Nutraceuticals containing grape leaves and/or seeds of the Vitacea *Vitis vinifera* L. can further claim physiological effects on humans. Indeed, their antioxidant activity, as well as their ability to reduce cardiovascular disease (CVD) risk by improving microcirculation and promoting homeostasis of the cardiovascular system, has been scientifically proven [22]. Similarly, nutraceuticals can contain fruits, seeds, buds, cortex ex radicibus of the Rosaceae *Malus domestica* B. and *Malus pumila* M., the consumption of which improves gastrointestinal transit and modulates the intestinal absorption of nutrients [22].

The list of botanicals allowed in nutraceuticals, however, does not have any legal force, and EFSA itself advices developers and producers to be responsible for the safety of their products, that must comply with the general requirements set out in the general food law [22]. Moreover, while mentioning seeds, leaves and other bioproducts of food processing, the list does not clearly refer to the use of agri-food waste as source for preparation of such nutraceuticals. At least two aspects of bio-waste should invite producers to confirm each time the safety of new nutraceutical products. The first relates to the chemical complexity of the biomass used as starting material, that could unexpectedly release chemical species in the final product. The second relates to the safety of the whole phytocomplex, the ensemble of chemical components present in the nutraceutical. Regulation advices to focus toxicity studies on each specific constituent of the nutraceutical [23]. However, as a consequence of possible synergistic or antagonistic effects, the pharmacokinetic parameters, bioavailability, bioaccessibility, bioactivity as well as overall toxicity of the whole product could be different from that of its components. As a consequence, the safety of individual substances cannot be used to draw general conclusions on whole extracts and botanical preparations, whose safety must thus be confirmed.

In 2009, the EFSA published the Guideline entitled “Safety assessment of botanicals and botanical preparations intended for use as ingredients in food supplements” [22]. The suggested approach to assess safety divides the evaluation into core areas and includes the assessment of mutagenicity of the food supplement by means of the Ames test (Organization for Economic Cooperation and Development (OECD) guideline 471) [24,25,26,27]. The same assesment and test is suggested and requested by other regulatory advisory bodies (e.g., European Chemicals Agency; UK Committee on Mutagenicity; the US Environmental Protection Agency).

The Ames test identifies and measures mutations occuring in bacterial genome upon exposure to a test chemical. DNA damage can be considered a surrogate endpoint for carcinogenicity [28,29], since the latter occurs in mammals as a consequence of an accumulation of a series of mutations. The test exposes five bacterial strains (*S. typhimurium* and *E. coli*) to a test substance. The bacterial strains used in the Ames test present mutations in genes needed for the synthesis of a specific amino acid (His in Salmonella and Trp in *E. coli*) [24,28]. Differently from wt strains, these bacteria lose auxotrophy for that amino acid and cannot grow in its absence. Furthermore, the bacterial strains present the rfa mutation, making their cell membrane more permeable to large molecules, and a deletion of the uvrB gene, abolishing the excision repair process, resulting in an increased sensitivity to mutagenic agents [24]. To identify chemicals aquiring genotoxicity upon in vivo metabolization, substances are tested with and without a metabolic activation system derived from rodent liver microsomes (called S9). Genotoxic chemicals revert the mutated gene sequence of the bacterial strain to the wt sequence allowing the growth of revertant colonies in the absence of amino acids [24].

Here, we use the Ames test to assess the mutagenicity of three nutraceuticals obtained from agri-food waste biomasses. The first phytocomplex tested is an extract of a grape pomace from *Vitis vinifera* cv ‘Aglianico’. This extract is used to prepare ‘Taurisolo^®^’, an antioxidant able to reduce serum levels of the cardiovascular risk factor markers oxidized-low-density lipoprotein (LDL) and Trimethylamine N-oxide (TMAO) in humans and in rodents [30,31,32], and improve microcirculation.

The other two nutraceuticals tested contain extracts of two italian apple cultivar, namely *Malus pumila* M. cv ‘Annurca’ and *Malus domestica* B. cv ‘Limoncella’. *Malus pumila* Miller cv. Annurca is a widespread apple and accounts for 5% of Italian apple production. It is listed as a Protected Geographical Indication (PGI) product from the European Council (Commission Regulation (EC) No. 417/2006)). Annurca Apples are used to prepare ‘AnnurComplex^®^’, a nutraceutical able to reduce serum cholesterol levels, LDL and lipid uptake; reduce cardiovascular disease (CVD) risk; and promote hair growth in humans [33,34,35,36,37]. *Malus domestica* cv ‘Limoncella’ is a juicy and aromatic variety of apple, known since ancient Roman times. Limoncella Apple extract presents high antioxidant activity and it has been shown to reduce colon inflammation and to act as potent inhibitor (in vitro and ex vivo) [38] of the Wingless-related integration site (WNT)-β catenin pathway, a signaling cascade linked to inflammation, oxidative stress, cell proliferation and cancerogenesis.

By means of the Ames test, we here show that the three nutraceuticals are not mutagenic. Our results support their safety and prove that the use of apple and wine waste product to produce polyphenolic mixtures does not enrich the final nutraceuticals of byproducts and secondary metabolites endowed with mutagenic potential.

## 2. Materials and Methods

### 2.1. Nutraceuticals

#### 2.1.1. Taurisolo^®^

Taurisolo is a nutraceutical supplement consisting of a polyphenol extract obtained from *Vitis Vinifera* cv ‘Aglianico’ grapes, collected in Montemarano (Avellino, Italy, Coordinates: 40°54′58″ N 14°59′54″ E) during the autumn harvest. The Department of Pharmacy of University of Naples Federico II (Naples, Italy), provided an initial supplement formulation, that was then produced in large scale by MB-Med Company (Turin, Italy). To produce the polyphenol extract, grapes were extracted with water (50 °C). The extract was then filtered and concentrated to finally undergo a spray-drying process with maltodextrins as support (40–70%) to obtain a fine microencapsulated powder. Taurisolo contains (μg/g): *Caffeic Acid* 26.42 ± 1.79; *Ferulic Acid* 11.78 ± 4.01; *Gallic Acid* 126.21 ± 57.3; *p-Coumaric acid* 18.81 ± 2.31; *Syringic acid* 368.95 ± 7.63; *Catechin* 3356.60 ± 68.41; *Epicatechin* 800.01 ± 32.2; *Procyanidin B1* 41.71 ± 3.25; *Procyanidin B2* 315.34 ± 8.92; *Procyanidin C1* 36.65 ± 3.24; *Quercetin* 27.25 ± 5.96; *Resveratrol* 10.30 ± 1.22; *Rutin* 15.78 ± 3.21.

#### 2.1.2. AnnurComplex^®^ and Limoncella Apple Extract

*Malus pumila* M. cv ‘Annurca’ apples were provided from ‘Consorzio Mela Annurca Campana Protected geographical indication (PGI)’. Annurca apples do not mature on the plant but complete their maturation upon harvesting and after a reddening period lasting ~30 days. *Malus domestica* B. cv ‘Limoncella’ apples were collected in October when fruits had just been harvested in Castelvetere del Calore (Avellino, Italy, Coordinates: 40°55′47″ N 14°59′13″ E). To produce AnnurComplex and Limoncella Apple Extract, 2nd-best Annurca and Limoncella apples have been used, respectively. Apples have been extracted with water, and the obtained solution was filtered, centrifuged, and concentrated. The extract underwent a spray-drying process with maltodextrins as support to obtain a fine microencapsulated powder. The supplements AnnurComplex and Limoncella Apple Extract were formulated by the Department of Pharmacy, University of Naples “Federico II” (Naples, Italy), and large-scale production was then accomplished by MB-Med Company (Turin, Italy). AnnurComplex contains (mg/100 g): *Chlorogenic acid* 5.48 ± 0.15; *Catechin* 0.52 ± 0.01; *Phlorizin* 3.88 ± 0.23; *Procyanidin B2* 2.15 ± 0.08; *Quercetin* 0.540 ± 0.002, *Rutin* 1.43 ± 0.05. Limoncella Apple Extract contains (mg/100 g): *Chlorogenic acid* 8.600 ± 0.002; *Catechin* 1.4 ± 0.1; *Phlorizin* 7.00 ± 0.53; *Procyanidin B2* 2.15 ± 0.09; *Quercetin* 1.200 ± 0.006, *Rutin* 2.600 ± 0.004.

### 2.2. Ames Test

Ames test was performed following the guidelines of OECD 471 [25,39,40] as described in Appendix A of this manuscript.

## 3. Results

Table A1, Table A2, Table A3, Table A4, Table A5, Table A6, Table A7, Table A8, Table A9, Table A10, Table A11, Table A12, Table A13, Table A14 and Table A15 (see Appendix B) present the raw data obtained performing the Ames test on the three nutraceuticals. Each table shows the number of revertants per plate, measured in three replicates, their means, the standard deviation (sd) observed in *S. typhimurium* strains TA98, TA100, TA1535, TA1537 and in the *E. coli* strain WP2 trp UvrA upon treatment with different doses of Taurisolo (Table A1, Table A2, Table A3, Table A4 and Table A5), AnnurComplex (Table A6, Table A7, Table A8, Table A9 and Table A10) and Limoncella Apple Extract (Table A11, Table A12, Table A13, Table A14 and Table A15). Treatments were performed in the presence (+S9) and in the absence (−S9) of metabolic activation.

Raw data are summarized in Table 1, Table 2 and Table 3, where the mutagenic index of Taurisolo (Table 1), AnnurComplex (Table 2) and Limoncella Apple Extract (Table 3) observed in the different bacterial strains are compared to known genotoxic substances. The mutagenic index is the ratio between the average number of revertants per plate measured upon incubation with the test nutraceutical and the average number of revertants per plate measured upon incubation with the negative (solvent) control. A chemical can be considered mutagenic when a two-fold increase in mutagenic index is observed upon treatment with at least one of the tested concentrations.

As shown in Table 1, Table 2 and Table 3, no mutagenicity was detected for any of the three nutraceuticals obtained from agri-food biowaste, both in the presence or in the absence of metabolic activation.

## 4. Discussion

The perception that food bio-waste is going to have a huge impact on human health is rapidly growing in modern society. The increasing demand for products of natural origin is exposing air, lands and waters to an unprecedented risk of pollution, with anticipated consequence on the whole ecosystem. Nowadays, agri-food biowaste, owing to their peculiar composition, is being used as starting material for sustainable production of pure chemicals and botanicals to be used in the pharmaceutical industry. Thus, while the processing of grapes and apples, the most cultivated fruits in the Mediterranean basin, are responsible for a large amount of bio-waste [5], the same can be reused for the production of nutraceuticals and food supplements, ultimately contributing to making this biomass recyclable and renewable [6].

By virtue of the peculiar nature of the agri-food biowaste used as starting material for preparation of these nutraceuticals, developers and producers must assess the safety of their products, in compliance with EFSA regulation [22,25]. During the last decades, the non-mutagenicity of several food components (including polyphenols) has been confirmed by means of the Ames test. For a few of them, including wine and apple components quercetin [41], kaempferol [42] caffeic and chlorogenic acid [43], the scientific community is still debating on their mild in vitro genotoxicity. It must be mentioned, however, that these reports tested the genotoxicity of pure polyphenols and not of phytocomplexes. Since the mutagenic activity of individual substances can be altered by the presence of other components of the mixture, their safety (or the unsafety) cannot be thus used to deduce that of a whole botanical preparation. Furthermore, considering that the chemical composition of agri-food biowaste can be influenced by several parameter (agro-geographic factors, type of cultivar, harvesting time, extraction protocol), the genotoxic potential must be assessed for each new nutraceutical formulation.

Genotoxic assessment appears necessary especially for food supplements and nutraceuticals containing *Vitis vinifera*. The scientific reports assessing the genotoxic potential of phytocomplexes gave indeed controversial results in terms of safety and mutagenicity of grape-derived ingredients. In 1982 and 1984 Stoltz and colleagues [44,45] analyzed the mutagenic potential of a wide variety of food and food products by means of the Ames test. In their surveys, components of polar fractions of raw grapes and grape juice demonstrated potent mutagenic activity, both in the presence and absence of metabolic activation. The work of Stoltz was followed by Patrineli et al. [46], who showed in 1996 a potent mutagenic activity of unfermented white grape juice. The same group suggested that this mutagenic potential of grape juice could be attributed, at least in part, to reactive oxygen species production, which may emanate from the one-electron reduction of quinoid structures present in grape juice [47].

Safety assessment of a grape extracts was assessed in 2002 by Yamakoshi et al. [48], who tested the genotoxic potential of proanthocyanidin-rich extract prepared from grape seeds of *Vitis vinifera* L. The authors did not measure an increase in the number of revertant colonies in the Ames test, either in the presence or absence of S-9 mix, claiming the non-genotoxicity of the extract. To our knowledge, the first report of the genotoxic assessment of a polyphenols-rich extract obtained from red grape pomace appeared in 2011, in a report published by Lluis et al. [49]. In their work, the authors attribute to their grape pomace extract a weak genotoxic activity. The grape pomace extract was shown to be mutagenic for only one of the tested strain (*S. typhimurium* TA1537) at the highest dose tested (5 mg/plate).

Here, we show that, following Ames test procedure, Taurisolo does not show signs of mutagenicity. It is hard to compare the results obtained by Lluis and colleagues [49] with those obtained here on Taurisolo. The difference in the outcome might be ascribed to a difference in the cultivar used as starting material, as well as the procedure used to produce the grape pomace extracts, (extraction in water and spray drying for Taurisolo vs. extraction in 50% ethanol followed by chromatographic enrichment of polyphenols for the extract of Lluis et al. [49]).

Regarding apple extracts obtained from agri-food biowaste, the closest food supplements to AnnurComplex and Limoncella Apple Extract has been described by Shoij et al. [19]. In their manuscript, the authors described the genotoxic activity of a polyphenol-rich extract produced from unripe apples. Assayed by means of Ames test, their extract showed a slight increase in the number of revertants at the dose of 2.5 mg/plate on the *S. typhimurium* TA98 strain without metabolic activation. However, none of the other bacterial strains tested (TA100, TA1535, TA1537, WP2) showed an increase in the number of revertants, with or without S9 mixture, at doses up to 5.0 mg/plate. Here, we show that AnnurComplex and Limoncella Apple Extract did not show sign of mutagenicity. The difference between the results may, again, be attributed to the different cultivar used to produce the supplements as well as the procedures followed (extraction in water and spray drying for AnnurComplex and Limoncella Apple Extract vs. treatment with pectolytic enzyme followed by chromatographic enrichment of polyphenols for the apple extract described by Shoij et al. [19].

## 5. Conclusions

The use of agri-food biowaste as starting material to produce nutraceuticals, food supplements and fortified foods can increase the amount of renewable and recyclable biomass, ultimately lowering the environmental impact caused by the high demand for biological products. The safety of the resulting nutraceuticals must, however, always be assessed. The three nutraceuticals Taurisolo, Annutricomplex and Limoncella Apple Extract obtained from grape pomace of *Vitis vinifera* L. cv ‘Aglianico’, *Malus pumila* M. cv ‘Annurca’ and *Malus domestica* B. cv ‘Limoncella’, respectively, were all assayed by means of the Ames tests and resulted to be non-mutagenic.

## Figures and Tables

**Table 1:** Mutagenicity of Taurisolo.

Mean Revertants										
		−S9					+S9			
**mg/plate**	**TA98**	**TA100**	**TA1535**	**TA1537**	**E.coli WP2 Uvr A**	**TA98**	**TA100**	**TA1535**	**TA1537**	***E.coli* WP2 Uvr A**
Negative control	26 ± 2.6	161.3 ± 10.2	15.6 ± 3.2	23.6 ± 3	35.6 ± 5.6	43.3 ± 4.7	217.3 ± 12.4	19 ± 6	19 ± 2	39 ± 2
0.0016	26 ± 5.1	184 ± 7.9	13 ± 1.7	22.6 ± 3.7	33.6 ± 2.3	50.3 ± 3	222.6 ± 5.8	22 ± 2.6	18.3 ± 2.3	44.6 ± 2.5
0.005	29.6 ± 4.7	202.3 ± 24.4	19 ± 2.6	23	32 ± 2	41.3 ± 2.8	230.6 ± 14.4	26.6 ± 0.5	25 ± 1.7	46 ± 3.4
0.016	32.3 ± 4.5	194 ± 20	20.6 ± 2.5	25 ± 3.6	37 ± 4.3	40 ± 2.6	224 ± 12.2	29.3 ± 1.5	26.6 ± 4.9	50.6 ± 3.7
0.05	28.3 ± 7.5	231 ± 10	22.6 ± 1.5	27 ± 2	41.3 ± 4.5	50 ± 3	219 ± 1	27 ± 4	24.6 ± 2.8	54 ± 2.6
0.16	29 ± 2	200.3 ± 18.6	19.6 ± 3	31.3 ± 1.1	40.3 ± 2.8	45.3 ± 2.8	211 ± 13.3	20.3 ± 0.5	23.6 ± 2	53.3 ± 1.1
0.5	31.3 ± 3	189.3 ± 13.6	21.6 ± 1.1	29.6 ± 6	39 ± 1.7	44.3 ± 3	215 ± 6.2	20.6 ± 0.5	31.6 ± 2	52 ± 3.4
1.6	32 ± 4	194 ± 25	19.6 ± 3	37 ± 2.6	40.3 ± 2.3	44.3 ± 6	241 ± 23.8	26 ± 3.6	31 ± 4.3	54.3 ± 5.5
5	32.6 ± 4.7	217 ± 12.1	27.6 ± 2.5	30.3 ± 0.5	44.6 ± 6.4	47 ± 6	232.6 ± 15.3	24.3 ± 2.3	24.6 ± 1.1	52 ± 2
Positive Control	1159.6 ± 69.6	1624 ± 54.1	212.3 ± 5.5	204.6 ± 9	182.6 ± 3	1357.6 ± 51.7	1751.6 ± 10.5	212.6 ± 2.5	234.3 ± 14.1	238.6 ± 11.3
**Mutagenic Index**		−S9					+S9			
**mg/plate**	**TA98**	**TA100**	**TA1535**	**TA1537**	**E.coli WP2 Uvr A**	**TA98**	**TA100**	**TA1535**	**TA1537**	***E.coli* WP2 Uvr A**
Negative control	1.0 ± 0.1	1.0 ± 0.1	1.0 ± 0.2	1.0 ± 0.1	1.0 ± 0.1	1.0 ± 0.1	1.0 ± 0.0	1.0 ± 0.3	1.0 ± 0.1	1.0 ± 0.0
0.0016	1.0 ± 0.1	1.1 ± 0.1	0.8 ± 0.1	1.0 ± 0.1	0.9 ± 0.1	1.2 ± 0.1	1.0 ± 0.0	1.2 ± 0.2	1.0 ± 0.1	1.1 ± 0.1
0.005	1.1 ± 0.1	1.3 ± 0.1	1.2 ± 0.2	1.0 ± 0.1	0.9 ± 0.1	1.0 ± 0.1	1.1 ± 0.1	1.4 ± 0.3	1.3 ± 0.1	1.2 ± 0.1
0.016	1.2 ± 0.1	1.2 ± 0.1	1.3 ± 0.2	1.1 ± 0.1	1.0 ± 0.1	0.9 ± 0.1	1.0 ± 0.0	1.5 ± 0.3	1.4 ± 0.2	1.3 ± 0.1
0.05	1.1 ± 0.2	1.4 ± 0.1	1.4 ± 0.2	1.1 ± 0.1	1.2 ± 0.1	1.2 ± 0.1	1.0 ± 0.0	1.4 ± 0.3	1.3 ± 0.1	1.4 ± 0.1
0.16	1.1 ± 0.1	1.2 ± 0.1	1.3 ± 0.2	1.3 ± 0.1	1.1 ± 0.1	1.0 ± 0.1	1.0 ± 0.0	1.1 ± 0.2	1.2 ± 0.1	1.4 ± 0.0
0.5	1.2 ± 0.1	1.2 ± 0.1	1.4 ± 0.2	1.3 ± 0.2	1.1 ± 0.1	1.0 ± 0.1	1.0 ± 0.0	1.1 ± 0.2	1.7 ± 0.1	1.3 ± 0.1
1.6	1.2 ± 0.1	1.2 ± 0.1	1.4 ± 0.2	1.6 ± 0.1	1.1 ± 0.1	1.0 ± 0.1	1.1 ± 0.1	1.4 ± 0.3	1.6 ± 0.2	1.4 ± 0.1
5	1.3 ± 0.1	1.3 ± 0.1	1.8 ± 0.2	1.3 ± 0.1	1.3 ± 0.2	1.1 ± 0.1	1.1 ± 0.1	1.3 ± 0.2	1.3 ± 0.1	1.3 ± 0.0
Positive Control	44.6 ± 3.0	10.1 ± 0.4	13.6 ± 1.6	8.6 ± 0.7	5.1 ± 0.5	31.3 ± 2.1	8.1 ± 0.3	11.2 ± 2.1	12.3 ± 0.9	6.1 ± 0.2

Mean revertants per plate and mutagenic index measured in bacterial strains TA98, TA100 and TA1535 and 1537 and WP2 treated with Taurisolo at various doses, with (+S9) or without (−S9) metabolic activation. Negative controls consisted of 100 μL water. Positive controls consisted: for *S. typhimurium* TA100 NaN3 (−S9) and BAP (+S9); for *S. typhimurium* TA98 2NF (−S9) and BAP (+S9); for *S. typhimurium* TA1535 NaN3 (−S9) and 2AA (+S9); for *S. typhimurium* TA1537 9AC (−S9) and 2AA (+S9); for *E. coli* WP2 trp UvrA NQO (−S9) and 2AA (+S9).

**Table 2:** Mutagenicity of AnnurComplex.

Mean Revertants										
		−S9					+S9			
**mg/plate**	**TA98**	**TA100**	**TA1535**	**TA1537**	***E.coli* WP2 Uvr A**	**TA98**	**TA100**	**TA1535**	**TA1537**	***E.coli* WP2 Uvr A**
Negative control	28 ± 4.5	177.3 ± 8.9	13 ± 1.7	18.6 ± 1.1	35.3 ± 2.3	36.6 ± 5.5	199 ± 15.3	18 ± 1	19 ± 1	39.3 ± 1.1
0.0016	25 ± 3.4	177.3 ± 12.4	18.3 ± 2.8	22.6 ± 3	34.3 ± 3.5	35 ± 1.7	211 ± 5.2	21 ± 2	22.3 ± 1.1	46.3 ± 1.1
0.005	28.3 ± 4.7	207.6 ± 9.2	16.6 ± 5	25.3 ± 2.3	31.3 ± 2.5	39.6 ± 3	216.6 ± 12.2	24.6 ± 2	25.3 ± 1.5	46.3 ± 3
0.016	31.6 ± 2	196.3 ± 12.6	18 ± 3.6	25.6 ± 4	36.3 ± 9.7	39.3 ± 1.1	215.3 ± 10	29.3 ± 5.1	27.6 ± 4.5	51.3 ± 1.1
0.05	26.3 ± 1.1	220 ± 12.5	20 ± 1.7	31.3 ± 2.3	39.6 ± 2.3	43.3 ± 2.3	215.6 ± 1.1	24.6 ± 4.9	22.6 ± 2.8	50.6 ± 0.5
0.16	32.6 ± 0.5	231.3 ± 33.6	22.3 ± 5.6	32 ± 5.3	36.3 ± 2.3	44.6 ± 5.7	211.3 ± 19.8	22.3 ± 5.5	23.6 ± 2.3	46 ± 4.5
0.5	36.3 ± 1.5	222.3 ± 12.5	19.3 ± 2.5	30 ± 1	33.3 ± 5	40.6 ± 0.5	222.6 ± 9	21	30.6 ± 3.5	49 ± 2
1.6	27.3 ± 6.5	194.3 ± 16.5	23.6 ± 0.5	28.6 ± 2	32.6 ± 4	41.3 ± 3	226.6 ± 12.5	22.6 ± 1.5	30 ± 2	51.3 ± 1.5
5	36.6 ± 2.3	217 ± 8.7	25.3 ± 1.1	34 ± 2	37.6 ± 7.5	34.6 ± 3	229.3 ± 0.5	28 ± 3	28.6 ± 3	44 ± 3.6
Positive Control	1219.3 ± 35.7	1650 ± 24	214 ± 6.5	200.3 ± 11.3	165.3 ± 14	1316.6 ± 32.1	1745.3 ± 9.4	215.6 ± 10.5	231.6 ± 7.3	237.6 ± 21.2
**Mutagenic Index**		−S9					+S9			
**mg/plate**	**TA98**	**TA100**	**TA1535**	**TA1537**	***E.coli* WP2 Uvr A**	**TA98**	**TA100**	**TA1535**	**TA1537**	***E.coli* WP2 Uvr A**
Negative control	1.0 ± 0.1	1.0 ± 0.1	1.0 ± 0.1	1.0 ± 0.1	1.0 ± 0.1	1.0 ± 0.0	1.0 ± 0.1	1.0 ± 0.0	1.0 ± 0.0	1.0 ± 0.0
0.0016	0.9 ± 0.1	1.0 ± 0.0	1.4 ± 0.2	1.2 ± 0.1	1.0 ± 0.1	1.0 ± 0.0	1.1 ± 0.0	1.2 ± 0.1	1.2 ± 0.1	1.2 ± 0.0
0.005	1.0 ± 0.1	1.2 ± 0.1	1.3 ± 0.2	1.4 ± 0.1	0.9 ± 0.1	1.1 ± 0.0	1.1 ± 0.1	1.4 ± 0.1	1.3 ± 0.1	1.2 ± 0.0
0.016	1.1 ± 0.1	1.1 ± 0.1	1.4 ± 0.2	1.4 ± 0.1	1.0 ± 0.2	1.1 ± 0.1	1.1 ± 0.1	1.6 ± 0.2	1.5 ± 0.1	1.3 ± 0.0
0.05	0.9 ± 0.1	1.2 ± 0.0	1.5 ± 0.1	1.7 ± 0.1	1.1 ± 0.1	1.2 ± 0.1	1.1 ± 0.0	1.4 ± 0.2	1.2 ± 0.1	1.3 ± 0.0
0.16	1.2 ± 0.1	1.3 ± 0.1	1.7 ± 0.3	1.7 ± 0.2	1.0 ± 0.1	1.2 ± 0.1	1.1 ± 0.1	1.2 ± 0.2	1.2 ± 0.1	1.2 ± 0.1
0.5	1.3 ± 0.1	1.3 ± 0.1	1.5 ± 0.2	1.6 ± 0.1	0.9 ± 0.1	1.1 ± 0.1	1.1 ± 0.1	1.2 ± 0.0	1.6 ± 0.1	1.2 ± 0.0
1.6	1.0 ± 0.1	1.1 ± 0.1	1.8 ± 0.1	1.5 ± 0.1	0.9 ± 0.1	1.1 ± 0.1	1.1 ± 0.1	1.3 ± 0.1	1.6 ± 0.1	1.3 ± 0.0
5	1.3 ± 0.1	1.2 ± 0.1	1.9 ± 0.2	1.8 ± 0.1	1.1 ± 0.1	0.9 ± 0.0	1.2 ± 0.1	1.6 ± 0.1	1.5 ± 0.1	1.1 ± 0.1
Positive Control	43.5 ± 3.2	9.3 ± 0.4	16.5 ± 1.3	10.7 ± 0.5	4.7 ± 0.3	35.9 ± 0.3	8.8 ± 0.4	12.0 ± 0.5	12.2 ± 0.4	6.0 ± 0.3

Mean revertants per plate and mutagenic index measured in bacterial strains TA98, TA100 and TA1535 and 1537 and WP2 treated with AnnurComplex at various doses, with (+S9) or without (−S9) metabolic activation. Negative controls consisted of 100 μL water. Positive controls consisted: for *S. typhimurium* TA100 NaN3 (−S9) and BAP (+S9); for *S. typhimurium* TA98 2NF (−S9) and BAP (+S9); for *S. typhimurium* TA1535 NaN3 (−S9) and 2AA (+S9); for *S. typhimurium* TA1537 9AC (−S9) and 2AA (+S9); for *E. coli* WP2 trp UvrA NQO (−S9) and 2AA (+S9).

**Table 3:** Mutagenicity of Limoncella Apple Extract.

Mean Revertants										
		−S9					+S9			
**mg/plate**	**TA98**	**TA100**	**TA1535**	**TA1537**	***E.coli* WP2 Uvr A**	**TA98**	**TA100**	**TA1535**	**TA1537**	***E.coli* WP2 Uvr A**
Negative control	25.6 ± 1.5	168.6 ± 7	16 ± 5.2	20.6 ± 4.6	37.3 ± 1.1	43 ± 1	190.3 ± 10.9	22.3 ± 4.5	24 ± 4	40.6 ± 3
0.0016	31 ± 2.6	177.6 ± 1.5	19.6 ± 2.5	26.3 ± 0.5	35.6 ± 1.1	44.6 ± 2	210.3 ± 6.1	25 ± 2	25.6 ± 1.1	44.3 ± 2.3
0.005	27.3 ± 1.5	203 ± 10.5	19.6 ± 3.2	26.6 ± 2.5	34.3 ± 5.5	37.6 ± 0.5	230.6 ± 3.5	26 ± 6	25.3 ± 1.1	45 ± 5.2
0.016	26 ± 1.7	195 ± 10.3	20.6 ± 1.1	29.3 ± 1.1	36.6 ± 5.7	41.3 ± 2.5	231.3 ± 6.8	24.6 ± 1.5	29.3 ± 0.5	50 ± 2
0.05	28 ± 3.6	213 ± 8.7	25.3 ± 6.1	31.6 ± 2	40.3 ± 2.5	46.3 ± 3	226.6 ± 7.5	25 ± 2.6	27.6 ± 3	50.6 ± 2.5
0.16	29 ± 3	197.6 ± 16.7	20.3 ± 2.5	30.6 ± 2	38 ± 1.7	42.6 ± 2.5	216.6 ± 13.6	22 ± 3.6	24.3 ± 1.5	47 ± 4
0.5	26 ± 1	195.6 ± 15	21 ± 2	32 ± 3.6	39.3 ± 0.5	36.3 ± 2.5	225.3 ± 16.5	24.6 ± 6.4	30.6 ± 1.5	47
1.6	34 ± 3	176 ± 3.4	23.6 ± 2.5	32.6 ± 2.5	41.3 ± 7.7	38.6 ± 2	227.6 ± 11	26.6 ± 0.5	29.6 ± 2.3	54 ± 6.5
5	34.3 ± 3.7	201.3 ± 7	25.3 ± 6.1	35.3 ± 2.5	42.6 ± 0.5	37 ± 3.4	230.6 ± 3	22.6 ± 1.1	27.3 ± 2.3	46 ± 2.6
Positive Control	1193.3 ± 63	1536.3 ± 37	204.3 ± 23.9	207.6 ± 10.6	187.6 ± 14.1	1320 ± 36	1711.3 ± 66.1	218 ± 13.8	221.6 ± 16	238.6 ± 9.6
**Mutagenic Index**		−S9					+S9			
**mg/plate**	**TA98**	**TA100**	**TA1535**	**TA1537**	***E.coli* WP2 Uvr A**	**TA98**	**TA100**	**TA1535**	**TA1537**	***E.coli* WP2 Uvr A**
Negative control	1.0 ± 0.0	1.0 ± 0.1	1.0 ± 0.3	1.0 ± 0.1	1.0 ± 0.1	1.0 ± 0.0	1.0 ± 0.1	1.0 ± 0.2	1.0 ± 0.1	1.0 ± 0.1
0.0016	1. 2 ± 0.1	1.1 ± 0.0	1.2 ± 0.3	1.3 ± 0.2	1.0 ± 0.0	1.0 ± 0.0	1.1 ± 0.0	1.1 ± 0.1	1.1 ± 0.1	1.1 ± 0.1
0.005	1.1 ± 0.1	1.2 ± 0.0	1.2 ± 0.3	1.3 ± 0.2	0.9 ± 0.1	0.9 ± 0.0	1.2 ± 0.0	1.2 ± 0.2	1.1 ± 0.1	1.1 ± 0.1
0.016	1.0 ± 0.1	1.2 ± 0.0	1.3 ± 0.3	1.4 ± 0.2	1.0 ± 0.1	1.0 ± 0.0	1.2 ± 0.0	1.1 ± 0.1	1.2 ± 0.1	1.2 ± 0.1
0.05	1.1 ± 0.1	1.3 ± 0.0	1.6 ± 0.4	1.5 ± 0.2	1.1 ± 0.0	1.1 ± 0.0	1.2 ± 0.0	1.1 ± 0.1	1.2 ± 0.1	1.2 ± 0.1
0.16	1.1 ± 0.1	1.2 ± 0.1	1.3 ± 0.3	1.5 ± 0.2	1.0 ± 0.0	1.0 ± 0.0	1.1 ± 0.1	1.0 ± 0.1	1.0 ± 0.1	1.2 ± 0.1
0.5	1.0 ± 0.0	1.2 ± 0.1	1.3 ± 0.3	1.5 ± 0.2	1.1 ± 0.0	0.8 ± 0.0	1.2 ± 0.1	1.1 ± 0.2	1.3 ± 0.1	1.2 ± 0.1
1.6	1.3 ± 0.1	1.0 ± 0.0	1.5 ± 0.3	1.6 ± 0.2	1.1 ± 0.1	0.9 ± 0.0	1.2 ± 0.1	1.2 ± 0.1	1.2 ± 0.1	1.3 ± 0.1
5	1.3 ± 0.1	1.2 ± 0.0	1.6 ± 0.4	1.7 ± 0.1	1.1 ± 0.0	0.9 ± 0.0	1.2 ± 0.0	1.0 ± 0.1	1.1 ± 0.1	1.1 ± 0.1
Positive Control	46.5 ± 2.1	9.1 ± 0.3	12.8 ± 2.6	10.0 ± 1.3	5.0 ± 0.2	30.7 ± 0.6	9.0 ± 0.4	9.8 ± 1.2	9.2 ± 1.0	5.9 ± 0.3

Mean revertants per plate and mutagenic index measured in bacterial strains TA98, TA100 and TA1535 and 1537 and WP2 treated with Limoncella Apple Extract at various doses, with (+S9) or without (−S9) metabolic activation. Negative controls consisted of 100 μL water. Positive controls consisted: for *S. typhimurium* TA100 NaN3 (−S9) and BAP (+S9); for *S. typhimurium* TA98 2NF (−S9) and BAP (+S9); for *S. typhimurium* TA1535 NaN3 (−S9) and 2AA (+S9); for *S. typhimurium* TA1537 9AC (−S9) and 2AA (+S9); for *E. coli* WP2 trp UvrA NQO (−S9) and 2AA (+S9).

## Appendix A

### Ames Test Procedure

*Chemicals and solvents.* Mitomycin C (Mit C. CAS Number 50-07-7. code 3514. LOT. NO F1318) was purchased from Santa Cruz Biotechnology (Santa Cruz, CA, USA). Ampicillin (code 26-810), Tetracycline (code 26-811), Crystal Violet (code 26-813), Benzo(a)pyrene (BAP. CAS 50-32-8. code 60-114.6. LOT. NO 8197BP), Sodium azide (NaN3, code 60-103.1), 2-aminoanthracene (2AA, code 60-107.21), 2-nitrofluorene (2-NF, code 60-111), 4-nitroquinoline-N-oxide (4-NQO, code 60-121.3), 9-aminoacridine (9AA, code 60-147.5) were all purchased from Trinova Biochem GmbH (Geissen. Germany) as well as MutazymeTM, 10%, Lyophilized Rat Liver S9 Mix (20 mL/vial, code 11-402L). When indicated chemicals were dissolved in sterile dimethyl sulfoxide (DMSO. CAS Number 67-68-5. JT Baker).

*Bacteriological Media.* Minimal Glucose Agar Plates: Agar (15 g/L), Vogel-Bonner salts (MgSO_4_ × 7H_2_O (200 mg/L), Citric Acid × H_2_O 2 g/L, KH_2_PO_4_ 10 g/L, (NH_4_) NaHPO_4_ × 4H_2_0 (3.5 g/L)), D-Glucose (4.0 g/L), pH 7.0.

Oxoid Agar Plates: Agar (15 g/L), Vogel-Bonner salts (MgSO_4_ × 7H_2_O (200 mg/L), Citric Acid × H_2_O 2 g/L, KH_2_PO_4_ 10 g/L. (NH_4_) NaHPO_4_ × 4H_2_O (3.5 g/L)), D-Glucose (2.0 g/L), Oxoid No.2 Broth (25 g/L), pH 7.0.

Top Agar: Agar (7 g/L), NaCl 5 g/L, L-Histidine HCl (10.4 mg/L), L-Tryptophan HCl (10.1 mg/L), D-Biotin (12.2 mg/L), pH 7.0. Media were steam sterilized at 15 lbs/sq for 20 min at 121 °C

*Bacterial Growth and storage.* Bacterial cultures were grown at the temperature of 37 °C in Pyrex flasks with 9 volume of air per volume of broth. Flasks were allocated in an incubator for prokaryotes on an orbital shaker shaking at the speed of 230 rpm. Cultures were grown up to late exponential phase (approximately 10^9^ cells per mL. Optical Density at λ = 600 nm of 1.0 ± 0.1). Titers were determined by plating a dilution 1:250,000 of the culture on Oxoid Agar Plates to then count the number of viable cells after 24 h of incubation. Long term storage of the bacterial strain was performed by keeping bacterial stocks at −80 °C in Oxoid No. 2 Broth supplemented with 20% of sterile Glycerol.

*Bacterial Strain.* The five bacterial strains *S. typhimurium* TA1535 (LOT. NO 5294D), *S. typhimurium* TA1537 (LOT NO. 5295D), *S. typhimurium* TA98 (LOT NO. 5293D), *S. typhimurium* TA100 (LOT NO. 5325D) and *E. coli* WP2 trp UvrA were purchased at Trinova Biochem (Giessen, Germany).

For *S. typhimurium* TA98 strain, phenotype confirmation was performed by growing an overnight culture in Oxoid No. 2 broth to then challenge 1–2 × 10^8^ of bacteria as follows: (a) the strain did not grow on agar minimal plate in the absence of L-His confirming the his^-^ phenotype; (b) the strain manifested zonal growth inhibition on agar minimal plate in the presence of L-His and of a 10 μg Crystal Violet disc. confirming the rfa phenotype; (c) the strain did grow on agar minimal plate containing L-His and a 2 μg Ampicillin disc confirming the presence of the R-factor plasmid; (d) the strain did not grow on agar minimal plate containing L-His, 2 μg Ampicillin and 1 μg Tetracycline disc confirm the absence of the pAQ1 plasmid; (e) the strain did not grow on agar minimal plate containing L-His and 0.2 μg Mitomycin disc confirming the uvrA/B phenotype and thus the absence of an active excision repair. The *S. typhimurium* TA98 strain yielded spontaneous revertant colony plate counts within the frequency ranges expected from the laboratory’s historical control data and within the range reported in the literature [23,28,40]. Mean revertant per plates: (water: number of colonies 26), Daunomycin (6 μg; number of colonies 954), ICR191 (1 μg; number of colonies 38), Mitomycin C (0.5 μg; number of colonies 10), NaN_3_ (1.5 μg; number of colonies 21 colonies).

For *S. typhimurium* TA100 strain, phenotype confirmation was performed by growing an overnight culture in Oxoid No. 2 broth to then challenge 1–2 × 10^8^ of bacteria as follows: (a) the strain did not grow on agar minimal plate in the absence of L-His confirming the his^-^ phenotype; (b) the strain manifested zonal growth inhibition on agar minimal plate in the presence of L-His and of a 10 μg Crystal Violet disc confirming the rfa phenotype; (c) the strain did grow on agar minimal plate containing L-His and 2 μg Ampicillin confirming the presence of the R-factor plasmid; (d) the strain did not grow on agar minimal plate containing L-His and of a 2 μg Ampicillin and 1 μg Tetracycline disc confirming the absence of the pAQ1 plasmid; (e) the strain did not grow on agar minimal plate containing L-His and a 0.2 μg Mitomycin disc confirming the uvrA/B phenotype and thus the absence of an active excision repair. The *S. typhimurium* TA100 strain yielded spontaneous revertant colony plate counts within the frequency ranges expected from the laboratory’s historical control data and within the range reported in the literature. Mean revertant per plates: (water: number of colonies 92), Daunomycin (6 μg; number of colonies 229), ICR191 (1 μg; number of colonies 117). Mitomycin C (0.5 μg; number of colonies 50), NaN_3_ (1.5 μg; number of colonies 558).

For *S. typhimurium* TA1535 strain, phenotype confirmation was performed by growing an overnight culture in Oxoid No. 2 broth to then challenge 1–2 × 10^8^ of bacteria as follows: (a) the strain did not grow on agar minimal plate in the absence of L-His confirming the his^-^ phenotype; (b) the strain manifested zonal growth inhibition on agar minimal plate in the presence of L-His and 10 μg Crystal Violet disc confirming the rfa phenotype; (c) the strain did not grow on agar minimal plate containing L-His and a 2 μg Ampicillin disc confirming the absence of the R-factor plasmid; (d) the strain did not grow on agar minimal plate containing L-His and a 2 μg Ampicillin and a 1 μg Tetracycline disc confirming the absence of the pAQ1 plasmid; (e) the strain did grow on agar minimal plate containing L-His and 0.2 μg Mitomycin disc confirming the uvrA/B phenotype and thus the absence of an active excision repair. The *S. typhimurium* TA1535 strain yielded spontaneous revertant colony plate counts within the frequency ranges expected from the laboratory’s historical control data and within the range reported in the literature. Mean revertant per plates: (water, number of colonies 6), Daunomycin (6 μg, number of colonies 5), ICR191 (1 μg, number of colonies 7), Mitomycin C (0.5 μg, number of colonies 0 colonies). NaN_3_ (1.5 μg, number of colonies 329 colonies).

For *S. typhimurium* TA1537 strain, phenotype confirmation was performed by growing an overnight culture in Oxoid No. 2 broth to then challenge 1–2 × 10^8^ of bacteria as follows: (a) the strain did not grow on agar minimal plate in the absence of L-His confirming the his^-^ phenotype; (b) the strain manifested zonal growth inhibition on agar minimal plate in the presence of L-His and a 10 μg Crystal Violet disc confirming the rfa phenotype; (c) the strain did not grow on agar minimal plate containing L-His and a 2 μg Ampicillin disc confirming the absence of the R-factor plasmid; (d) the strain did not grow on agar minimal plate containing L-HisAnd a 2 μg Ampicillin and 1 μg Tetracycline disc confirming the absence of the pAQ1 plasmid; (e) the strain did grow on agar minimal plate containing L-His and 0.2 μg Mitomycin disc confirming the uvrA/B phenotype and thus the absence of an active excision repair. The *S. typhimurium* TA1537 strain yielded spontaneous revertant colony plate counts within the frequency ranges expected from the laboratory’s historical control data and within the range reported in the literature. Mean revertant per plates: water (number of colonies: 6), Daunomycin (6 μg, number of colonies: 10), ICR191 (1 μg, number of colonies: 60), Mitomycin C (0.5 μg, number of colonies: 1), NaN3 (1.5 μg, number of colonies: 10).

For *E. coli* WP2 trp UvrA strain, phenotype confirmation was performed by growing an overnight culture in Oxoid No. 2 broth to then challenge 1–2 × 10^8^ of bacteria as follows: (a) the strain did not grow on agar minimal plate in the absence of L-Trp confirming the trp^-^ phenotype; (b) the strain did not grow on agar minimal plate containing L-Trp and a 2 μg Ampicillin disc confirming the absence of the R-factor plasmid pKM101; (c) the strain did not grow on agar minimal plate containing L-Trp and a 0.2 μg Mitomycin disc confirming the UvrA/B phenotype and thus the absence of an active excision repair. The *E. coli* WP2 trp UvrA strain yield spontaneous revertant colony plate counts within the frequency ranges expected from the laboratory’s historical control data and within the range reported in the literature. Mean revertant per plates: water (number of colonies: 47), Methyl methanesulfonate (MMS) (2.5 μL, number of colonies: 528).

*Metabolic activation*. Metabolic activation of nutraceuticals was achieved by exogenous metabolization using S9 post-mitochondrial fraction. S9 (code 11-402L. LOT NO. 4026) prepared from livers of Sprague Dawley male rats treated with Aroclor 1254 (500 mg/Kg i.p.). Lyophilized S9 was purchased from Trinova Biochem already supplemented with glucose-6-phosphatedehydrogenase (180 mg/mL) Nicotinamide adenine dinucleotide phosphate (25 mg/mL), Potassium chloride (150 mM) mixed in the ratio 2:1:1:1. S9 was reconstituted in deionized water and stored at −80 °C. The protein concentration of S9, assayed with the Lowry Method, was 3.5 mg/mL. To prove S9 able to activate pro-mutagens, we measured the number of revertant colonies of TA98 and TA1535 strains growing in the presence of S9 and of ethidium bromide and cyclophosphamide. TA98 strain yielded 52 colonies in the presence of ethidium bromide and TA1535 yielded 430 colonies in the presence of cyclophosphamide, respectively. Dilution of S9, ranging from 0.6 to 10% were tested for their ability to activate benzo (a) pyrene and 2-aminoanthracene (2-AA) to metabolites mutagenic to TA100. The final concentration of S-9 fraction in the test system was 7% *v/v*. Cultures treated in the absence of S9 received an equivalent volume of 0.1 M phosphate Buffer pH 7.4 in place of S9 mix.

*Nutraceutical test conditions*. Taurisolo, AnnurComplex and Limoncella Apple Extract are highly soluble in water that was thus used as vehicle for all the experiments. Mother stocks of Nutraceuticals 50 mg/mL were freshly prepared in water. The recommended maximum test concentration for soluble non-cytotoxic substances is 5 mg/plate. None of the nutraceutical gave precipitation on the surface of the agar plate. However, the grape pomace stained the agar plate in a dark purple color.

Test dilutions were obtained by diluting mother stocks in water. We tested eight dilutions for each of the nutraceuticals (namely, 0.0016, 0.005, 0.016, 0.05, 0.16, 0.5, 1.6. and 5 mg/10 cm plate) (volume 100 μL). Up to 5 mg/plate and on Oxoid Agar Plates, none of the nutraceutical-induced growth inhibition of the bacterial strains here tested, confirming that in the range of dilution here assayed, all the tested nutraceuticals were not cytotoxic for the bacteria strains.

Negative controls consisted of 100 μL water. Positive controls consisted: for *S. typhimurium* TA100, NaN_3_ 1.25 μg/10 cm plate in the absence of S9 and BAP 6.0 μg/10 cm plate in the presence of S9; for *S. typhimurium* TA98, 2NF 2.0 μg/10 cm plate in the absence of S9 and BAP 6.0 μg/10 cm plate in the presence of S9; for *S. typhimurium* TA1535, NaN_3_ 1.25 μg/10 cm plate in the absence of S9 and 2AA 2.0 μg/10 cm plate in the presence of S9; for *S. typhimurium* TA1537, 9AC 50.0 μg/10 cm plate in the absence of S9 and 2AA 2 μg/10 cm plate in the presence of S9; for *E. coli WP2 trp UvrA,* NQO 1.0 μg/10 cm plate in the absence of S9 and 2AA 20.0 μg/10 cm plate in the presence of S9.

*Experimental Procedure*. We used the plate incorporation method. Briefly, 0.1 mL of test solutions (the appropriated amount of nutraceutical dissolved in 0.1 mL of water), 0.1 mL of fresh bacterial culture containing 10^8^ viable cells and either 0.5 mL of 0.1 M Phosphate buffer (pH 7.4) or 0.5 mL of S9 were mixed with 2.0 mL of Top Agar. For the assay with metabolic activation, 0.5 mL of metabolic activation mixture contained 7% post-mitochondrial fraction. The contents of each tube were mixed and poured over the surface of a minimal agar plate. The overlay agar was allowed to solidify before incubation. Experiments were performed in triplicates for each condition. Plates were incubated at 37 °C for 72 h. After the incubation period, the number of revertant colonies per plate was counted.

*Acceptance of the test.* Acceptance of the test was based on the following criteria: (a) all experimental conditions requested by OECD 471 were tested; (b) the results obtained for the negative control were consistent with the laboratory’s historical negative control database; (c) concurrent positive controls induced responses that were compatible with those generated in the laboratory’s historical positive control database and produced a statistically significant increase compared with the concurrent negative control.

## Appendix B

**Table A1:** Number of revertants/plate for *S. typhimurium* TA98 strain treated with Taurisolo.

		−S9				+S9		
mg/plate	Replicate1	Replicate2	Replicate3	Mean ± sd	Replicate1	Replicate2	Replicate3	Mean ± sd
Negative control	23	27	28	26 ± 2.6	38	45	47	43.3 ± 4.7
0.0016	23	23	32	26 ± 5.1	47	51	53	50.3 ± 3
0.005	26	35	28	29.6 ± 4.7	38	43	43	41.3 ± 2.8
0.016	37	28	32	32.3 ± 4.5	39	43	38	40 ± 2.6
0.05	37	24	24	28.3 ± 7.5	47	53	50	50 ± 3
0.16	27	29	31	29 ± 2	42	47	47	45.3 ± 2.8
0.5	32	28	34	31.3 ± 3	41	47	45	44.3 ± 3
1.6	36	32	28	32 ± 4	38	45	50	44.3 ± 6
5	29	38	31	32.6 ± 4.7	40	50	51	47 ± 6
Positive Control	1102	1140	1237	1159.6 ± 69.6	1300	1400	1373	1357.6 ± 51.7

Negative Control: water—100 μL/plate; Positive Control: 2-Nitro Fluorene (2.0 μg/plate) in the absence of S9 and Benzo(a)pyrene (6 μg/plate) in the presence of S9. Historical negative in the absence of S9: Range 22–57 (mean ± s.d. = 29 ± 8). Historical negative in the presence of S9: Range 20–53 (mean ± s.d. = 43 ± 13). Historical positive in the absence of S9: Range 1109–1363 (mean ± s.d. = 1214 ± 46). Historical positive in the presence of S9: Range 236–1348 (mean ± s.d. = 1321 ± 33).

**Table A2:** Number of revertants/plate for *S. typhimurium* TA100 strain treated with Taurisolo.

		−S9				+S9		
mg/plate	Replicate1	Replicate2	Replicate3	Mean ± sd	Replicate1	Replicate2	Replicate3	Mean ± sd
Negative control	150	170	164	161.3 ± 10.2	203	225	224	217.3 ± 12.4
0.0016	190	187	175	184 ± 7.9	216	225	227	222.6 ± 5.8
0.005	210	222	175	202.3 ± 24.4	214	240	238	230.6 ± 14.4
0.016	175	192	215	194 ± 20	215	238	219	224 ± 12.2
0.05	220	240	233	231 ± 10	220	219	218	219 ± 1
0.16	198	220	183	200.3 ± 18.6	197	215	223	211 ± 13.3
0.5	180	205	183	189.3 ± 13.6	222	210	213	215 ± 6.2
1.6	192	220	170	194 ± 25	215	262	246	241 ± 23.8
5	230	215	206	217 ± 12.1	240	243	215	232.6 ± 15.3
Positive Control	1572	1620	1680	1624 ± 54.1	1750	1742	1763	1751.6 ± 10.5

Negative Control: water—100 μL/plate; Positive Control Sodium Azide (1.25 μg/plate) in the absence of S9 and Benzo(a)pyrene (6 μg/plate) in the presence of S9. Historical negative in the absence of S9: Range 144–240 (mean ± s.d. = 195 ± 15). Historical negative in the presence of S9: Range 176–250 (mean ± s.d. = 211 ± 21). Historical positive in the absence of S9: Range 1428–1620 (mean ± s.d. = 1480 ± 80). Historical positive in the presence of S9: Range 1600–1923 (mean ± s.d. = 1693 ± 72).

**Table A3:** Number of revertants/plate for *S. typhimurium* TA1535 strain treated with Taurisolo.

		−S9				+S9		
mg/plate	Replicate1	Replicate2	Replicate3	Mean ± sd	Replicate1	Replicate2	Replicate3	Mean ± sd
Negative control	12	18	17	15.6 ± 3.2	22	23	12	19 ± 6
0.0016	12	12	15	13 ± 1.7	24	23	19	22 ± 2.6
0.005	20	16	21	19 ± 2.6	26	27	27	26.6 ± 0.5
0.016	18	21	23	20.6 ± 2.5	28	29	31	29.3 ± 1.5
0.05	21	24	23	22.6 ± 1.5	31	27	23	27 ± 4
0.16	19	17	23	19.6 ± 3	20	20	21	20.3 ± 0.5
0.5	21	21	23	21.6 ± 1.1	21	21	20	20.6 ± 0.5
1.6	24	23	21	19.6 ± 3	23	25	30	26 ± 3.6
5	30	28	25	27.6 ± 2.5	23	23	27	24.3 ± 2.3
Positive Control	206	215	216	212.3 ± 5.5	210	215	213	212.6 ± 2.5

Negative Control: water—100 μL/plate; Positive Control: Sodium Azide (1.25 μg/plate) in the absence of S9 and 2-aminoanthracene (2 μg/plate) in the presence of S9. Historical negative in the absence of S9: Range 12–45 (mean ± s.d. = 26 ± 5). Historical negative in the presence of S9: Range 17–35 (mean ± s.d. = 23 ± 9). Historical positive in the absence of S9: Range 195–240 (mean ± s.d. = 202 ± 36). Historical positive in the presence of S9: Range 222–286 (mean ± s.d. = 232 ± 26).

**Table A4:** Number of revertants/plate for *S. typhimurium* TA1537 strain treated with Taurisolo.

		−S9				+S9		
mg/plate	Replicate1	Replicate2	Replicate3	Mean ± sd	Replicate1	Replicate2	Replicate3	Mean ± sd
Negative control	21	23	27	23.6 ± 3	21	19	17	19 ± 2
0.0016	27	21	20	22.6 ± 3.7	17	17	21	18.3 ± 2.3
0.005	23	23	23	23	23	26	26	25 ± 1.7
0.016	26	28	21	25 ± 3.6	30	29	21	26.6 ± 4.9
0.05	29	27	25	27 ± 2	28	23	23	24.6 ± 2.8
0.16	32	30	32	31.3 ± 1.1	26	22	23	23.6 ± 2
0.5	24	29	36	29.6 ± 6	34	31	30	31.6 ± 2
1.6	36	35	40	37 ± 2.6	28	29	36	31 ± 4.3
5	30	30	31	30.3 ± 0.5	24	24	26	24.6 ± 1.1
Positive Control	198	215	201	204.6 ± 9	218	242	243	234.3 ± 14.1

Negative Control: water—100 μL/plate; Positive Control: 9-aminoacridine HCl (50.0 μg/plate) in the absence of S9 and 2-amino anthracene (2 μg/plate) in the presence of S9. Historical negative in the absence of S9: Range 19–40 (mean ± s.d. = 32 ± 10). Historical negative in the presence of S9: Range 18–43 (mean ± s.d. = 31 ± 6). Historical positive in the absence of S9: Range 187–250 (mean ± s.d. = 210 ± 42). Historical positive in the presence of S9: Range 223–270 (mean ± s.d. = 216 ± 34).

**Table A5:** Number of revertants/plate for *E. coli WP2 Uvr A* strain treated with Taurisolo.

		−S9				+S9		
mg/plate	Replicate1	Replicate2	Replicate3	Mean ± sd	Replicate1	Replicate2	Replicate3	Mean ± sd
Negative control	31	42	34	35.6 ± 5.6	39	41	37	39 ± 2
0.0016	31	35	35	33.6 ± 2.3	45	47	42	44.6 ± 2.5
0.005	30	32	34	32 ± 2	48	48	42	46 ± 3.4
0.016	35	42	34	37 ± 4.3	48	49	55	50.6 ± 3.7
0.05	37	41	46	41.3 ± 4.5	56	55	51	54 ± 2.6
0.16	37	42	42	40.3 ± 2.8	54	54	52	53.3 ± 1.1
0.5	40	40	37	39 ± 1.7	50	50	56	52 ± 3.4
1.6	39	39	43	40.3 ± 2.3	57	48	58	54.3 ± 5.5
5	42	52	40	44.6 ± 6.4	50	52	54	52 ± 2
Positive Control	180	182	186	182.6 ± 3	242	248	226	238.6 ± 11.3

Negative Control: water—100 μL/plate; Positive Control: 4-nitroquinoline-N-oxide (1.0 μg/plate) in the absence of S9 and 2 amino- anthracene (20 μg/plate) in the presence of S9. Historical negative in the absence of S9: Range 37–58 (mean ± s.d. = 45 ± 11). Historical negative in the presence of S9: Range 32–70 (mean ± s.d. = 44 ± 6). Historical positive in the absence of S9: Range 109–195 (mean ± s.d. = 177 ± 29). Historical positive in the presence of S9: Range 193–263 (mean ± s.d. = 210 ± 34).

**Table A6:** Number of revertants/plate for *S. typhimurium* TA98 strain treated with AnnurComplex.

		−S9				+S9		
mg/plate	Replicate1	Replicate2	Replicate3	Mean ± sd	Replicate1	Replicate2	Replicate3	Mean ± sd
Negative control	24	27	33	28 ± 4.5	31	37	42	36.6 ± 5.5
0.0016	21	27	27	25 ± 3.4	34	34	37	35 ± 1.7
0.005	32	30	23	28.3 ± 4.7	37	39	43	39.6 ± 3
0.016	30	31	34	31.6 ± 2	38	40	40	39.3 ± 1.1
0.05	25	27	27	26.3 ± 1.1	42	46	42	43.3 ± 2.3
0.16	32	33	33	32.6 ± 0.5	38	48	48	44.6 ± 5.7
0.5	35	38	36	36.3 ± 1.5	40	41	41	40.6 ± 0.5
1.6	27	34	21	27.3 ± 6.5	42	44	38	41.3 ± 3
5	34	38	38	36.6 ± 2.3	38	32	34	34.6 ± 3
Positive Control	1180	1250	1228	1219.3 ± 35.7	1280	1340	1330	1316.6 ± 32.1

Negative Control: water—100 μL/plate; Positive Control: 2-Nitro Fluorene (2.0 μg/plate) in the absence of S9 and Benzo(a)pyrene (6 μg/plate) in the presence of S9. Historical negative in the absence of S9: Range 22–57 (mean ± s.d. = 29 ± 8). Historical negative in the presence of S9: Range 20–53 (mean ± s.d. = 43 ± 13). Historical positive in the absence of S9: Range 1109–1363 (mean ± s.d. = 1214 ± 46). Historical positive in the presence of S9: Range 236–1348 (mean ± s.d. = 1321 ± 33).

**Table A7:** Number of revertants/plate for *S. typhimurium* TA100 strain treated with AnnurComplex.

		−S9				+S9		
mg/plate	Replicate1	Replicate2	Replicate3	Mean ± sd	Replicate1	Replicate2	Replicate3	Mean ± sd
Negative control	167	183	182	177.3 ± 8.9	182	203	212	199 ± 15.3
0.0016	184	185	163	177.3 ± 12.4	207	209	217	211 ± 5.2
0.005	212	214	197	207.6 ± 9.2	230	214	206	216.6 ± 12.2
0.016	194	210	185	196.3 ± 12.6	219	223	204	215.3 ± 10
0.05	221	232	207	220 ± 12.5	215	215	217	215.6 ± 1.1
0.16	202	224	268	231.3 ± 33.6	197	203	234	211.3 ± 19.8
0.5	235	210	222	222.3 ± 12.5	222	214	232	222.6 ± 9
1.6	196	210	177	194.3 ± 16.5	214	239	227	226.6 ± 12.5
5	223	221	207	217 ± 8.7	229	229	230	229.3 ± 0.5
Positive Control	1631	1677	1642	1650 ± 24	1756	1738	1742	1745.3 ± 9.4

Negative Control: water—100 μL/plate; Positive Control Sodium Azide (1.25 μg/plate) in the absence of S9 and Benzo(a)pyrene (6 μg/plate) in the presence of S9. Historical negative in the absence of S9: Range 144–240 (mean ± s.d. = 195 ± 15). Historical negative in the presence of S9: Range 176–250 (mean ± s.d. = 211 ± 21). Historical positive in the absence of S9: Range 1428–1620 (mean ± s.d. = 1480 ± 80). Historical positive in the presence of S9: Range 1600–1923 (mean ± s.d. = 1693 ± 72).

**Table A8:** Number of revertants/plate for *S. typhimurium* TA1535 strain treated with AnnurComplex.

		−S9				+S9		
mg/plate	Replicate1	Replicate2	Replicate3	Mean ± sd	Replicate1	Replicate2	Replicate3	Mean ± sd
Negative control	12	12	15	13 ± 1.7	18	17	19	18 ± 1
0.0016	17	17	22	18.3 ± 2.8	21	23	19	21 ± 2
0.005	16	12	22	16.6 ± 5	24	23	27	24.6 ± 2
0.016	17	15	22	18 ± 3.6	25	28	35	29.3 ± 5.1
0.05	19	19	22	20 ± 1.7	27	28	19	24.6 ± 4.9
0.16	27	16	24	22.3 ± 5.6	17	28	22	22.3 ± 5.5
0.5	19	17	22	19.3 ± 2.5	21	21	21	21
1.6	24	24	23	23.6 ± 0.5	24	21	23	22.6 ± 1.5
5	26	26	24	25.3 ± 1.1	25	28	31	28 ± 3
Positive Control	215	220	207	214 ± 6.5	214	227	206	215.6 ± 10.5

Negative Control: water—100 μL/plate; Positive Control: Sodium Azide (1.25 μg/plate) in the absence of S9 and 2-aminoanthracene (2 μg/plate) in the presence of S9. Historical negative in the absence of S9: Range 12–45 (mean ± s.d. = 26 ± 5). Historical negative in the presence of S9: Range 17–35 (mean ± s.d. = 23 ± 9). Historical positive in the absence of S9: Range 195–240 (mean ± s.d. = 202 ± 36). Historical positive in the presence of S9: Range 222–286 (mean ± s.d. = 232 ± 26).

**Table A9:** Number of revertants/plate for *S. typhimurium* TA1537 strain treated with AnnurComplex.

		−S9				+S9		
mg/plate	Replicate1	Replicate2	Replicate3	Mean ± sd	Replicate1	Replicate2	Replicate3	Mean ± sd
Negative control	18	20	18	18.6 ± 1.1	20	18	19	19 ± 1
0.0016	22	26	20	22.6 ± 3	23	21	23	22.3 ± 1.1
0.005	24	24	28	25.3 ± 2.3	25	27	24	25.3 ± 1.5
0.016	28	28	21	25.6 ± 4	32	28	23	27.6 ± 4.5
0.05	30	30	34	31.3 ± 2.3	26	21	21	22.6 ± 2.8
0.16	26	36	34	32 ± 5.3	21	25	25	23.6 ± 2.3
0.5	30	29	31	30 ± 1	31	34	27	30.6 ± 3.5
1.6	28	27	31	28.6 ± 2	30	32	28	30 ± 2
5	32	36	34	34 ± 2	26	32	28	28.6 ± 3
Positive Control	191	197	213	200.3 ± 11.3	240	229	226	231.6 ± 7.3

Negative Control: water—100 μL/plate; Positive Control: 9-aminoacridine HCl (50.0 μg/plate) in the absence of S9 and 2-amino anthracene (2 μg/plate) in the presence of S9. Historical negative in the absence of S9: Range 19–40 (mean ± s.d. = 32 ± 10). Historical negative in the presence of S9: Range 18–43 (mean ± s.d. = 31 ± 6). Historical positive in the absence of S9: Range 187–250 (mean ± s.d. = 210 ± 42). Historical positive in the presence of S9: Range 223–270 (mean ± s.d. = 216 ± 34).

**Table A10:** Number of revertants/plate for *E. coli WP2 Uvr A* strain treated with AnnurComplex.

		−S9				+S9		
mg/plate	Replicate1	Replicate2	Replicate3	Mean ± sd	Replicate1	Replicate2	Replicate3	Mean ± sd
Negative control	34	38	34	35.3 ± 2.3	38	40	40	39.3 ± 1.1
0.0016	31	34	38	34.3 ± 3.5	45	47	47	46.3 ± 1.1
0.005	29	31	34	31.3 ± 2.5	49	43	47	46.3 ± 3
0.016	28	47	34	36.3 ± 9.7	52	52	50	51.3 ± 1.1
0.05	37	41	41	39.6 ± 2.3	50	51	51	50.6 ± 0.5
0.16	39	35	35	36.3 ± 2.3	47	50	41	46 ± 4.5
0.5	38	28	34	33.3 ± 5	49	47	51	49 ± 2
1.6	37	29	32	32.6 ± 4	53	51	50	51.3 ± 1.5
5	41	29	43	37.6 ± 7.5	41	43	48	44 ± 3.6
Positive Control	161	181	154	165.3 ± 14	228	262	223	237.6 ± 21.2

Negative Control: water—100 μL/plate; Positive Control: 4-nitroquinoline-N-oxide (1.0 μg/plate) in the absence of S9 and 2 amino- anthracene (20 μg/plate) in the presence of S9. Historical negative in the absence of S9: Range 37–58 (mean ± s.d. = 45 ± 11). Historical negative in the presence of S9: Range 32–70 (mean ± s.d. = 44 ± 6). Historical positive in the absence of S9: Range 109–195 (mean ± s.d. = 177 ± 29). Historical positive in the presence of S9: Range 193–263 (mean ± s.d. = 210 ± 34).

**Table A11:** Number of revertants/plate for *S. typhimurium* TA98 treated with Limoncella Extract.

		−S9				+S9		
mg/plate	Replicate1	Replicate2	Replicate3	Mean ± sd	Replicate1	Replicate2	Replicate3	Mean ± sd
Negative control	26	27	24	25.6 ± 1.5	43	42	44	43 ± 1
0.0016	28	33	32	31 ± 2.6	43	44	47	44.6 ± 2
0.005	29	26	27	27.3 ± 1.5	38	37	38	37.6 ± 0.5
0.016	24	27	27	26 ± 1.7	39	41	44	41.3 ± 2.5
0.05	24	29	31	28 ± 3.6	43	47	49	46.3 ± 3
0.16	26	29	32	29 ± 3	43	40	45	42.6 ± 2.5
0.5	27	25	26	26 ± 1	34	36	39	36.3 ± 2.5
1.6	34	37	31	34 ± 3	41	37	38	38.6 ± 2
5	30	36	37	34.3 ± 3.7	39	33	39	37 ± 3.4
Positive Control	1121	1237	1222	1193.3 ± 63	1290	1310	1360	1320 ± 36

Negative Control: water—100 μL/plate; Positive Control: 2-Nitro Fluorene (2.0 μg/plate) in the absence of S9 and Benzo(a)pyrene (6 μg/plate) in the presence of S9. Historical negative in the absence of S9: Range 22–57 (mean ± s.d. = 29 ± 8). Historical negative in the presence of S9: Range 20–53 (mean ± s.d. = 43 ± 13). Historical positive in the absence of S9: Range 1109–1363 (mean ± s.d. = 1214 ± 46) Historical positive in the presence of S9: Range 236–1348 (mean ± s.d. = 1321 ± 33).

**Table A12:** Number of revertants/plate for *S. typhimurium* TA100 treated with Limoncella Extract.

		−S9				+S9		
mg/plate	Replicate1	Replicate2	Replicate3	Mean ± sd	Replicate1	Replicate2	Replicate3	Mean ± sd
Negative control	162	168	176	168.6 ± 7	184	184	203	190.3 ± 10.9
0.0016	178	179	176	177.6 ± 1.5	205	209	217	210.3 ± 6.1
0.005	195	215	199	203 ± 10.5	231	227	234	230.6 ± 3.5
0.016	189	207	189	195 ± 10.3	226	239	229	231.3 ± 6.8
0.05	217	219	203	213 ± 8.7	219	227	234	226.6 ± 7.5
0.16	187	217	189	197.6 ± 16.7	206	212	232	216.6 ± 13.6
0.5	197	210	180	195.6 ± 15	225	209	242	225.3 ± 16.5
1.6	174	180	174	176 ± 3.4	217	239	227	227.6 ± 11
5	195	200	209	201.3 ± 7	230	228	234	230.6 ± 3
Positive Control	1537	1499	1573	1536.3 ± 37	1635	1750	1749	1711.3 ± 66.1

Negative Control: water—100 μL/plate; Positive Control Sodium Azide (1.25 μg/plate) in the absence of S9 and Benzo(a)pyrene (6 μg/plate) in the presence of S9. Historical negative in the absence of S9: Range 144–240 (mean ± s.d. = 195 ± 15); Historical negative in the presence of S9: Range 176–250 (mean ± s.d. = 211 ± 21). Historical positive in the absence of S9: Range 1428–1620 (mean ± s.d. = 1480 ± 80). Historical positive in the presence of S9: Range 1600–1923 (mean ± s.d. = 1693 ± 72).

**Table A13:** Number of revertants/plate for *S. typhimurium* TA1535 treated with Limoncella Extract.

		−S9				+S9		
mg/plate	Replicate1	Replicate2	Replicate3	Mean ± sd	Replicate1	Replicate2	Replicate3	Mean ± sd
Negative control	10	20	18	16 ± 5.2	18	22	27	22.3 ± 4.5
0.0016	20	17	22	19.6 ± 2.5	23	27	25	25 ± 2
0.005	16	22	21	19.6 ± 3.2	20	26	32	26 ± 6
0.016	20	20	22	20.6 ± 1.1	26	25	23	24.6 ± 1.5
0.05	20	24	32	25.3 ± 6.1	24	28	23	25 ± 2.6
0.16	20	18	23	20.3 ± 2.5	21	19	26	22 ± 3.6
0.5	19	21	23	21 ± 2	22	20	32	24.6 ± 6.4
1.6	26	21	24	23.6 ± 2.5	27	27	26	26.6 ± 0.5
5	20	32	24	25.3 ± 6.1	24	22	22	22.6 ± 1.1
Positive Control	190	191	232	204.3 ± 23.9	210	210	234	218 ± 13.8

Negative Control: water—100 μL/plate; Positive Control: Sodium Azide (1.25 μg/plate) in the absence of S9 and 2-aminoanthracene (2 μg/plate) in the presence of S9. Historical negative in the absence of S9: Range 12–45 (mean ± s.d. = 26 ± 5). Historical negative in the presence of S9: Range 17–35 (mean ± s.d. = 23 ± 9). Historical positive in the absence of S9: Range 195–240 (mean ± s.d. = 202 ± 36). Historical positive in the presence of S9: Range 222–286 (mean ± s.d. = 232 ± 26).

**Table A14:** Number of revertants/plate for *S. typhimurium* TA1537 treated with Limoncella Extract.

		−S9				+S9		
mg/plate	Replicate1	Replicate2	Replicate3	Mean ± sd	Replicate1	Replicate2	Replicate3	Mean ± sd
Negative control	18	18	26	20.6 ± 4.6	20	28	24	24 ± 4
0.0016	26	26	27	26.3 ± 0.5	25	25	27	25.6 ± 1.1
0.005	24	27	29	26.6 ± 2.5	26	26	24	25.3 ± 1.1
0.016	30	30	28	29.3 ± 1.1	30	29	29	29.3 ± 0.5
0.05	31	34	30	31.6 ± 2	25	27	31	27.6 ± 3
0.16	29	30	33	30.6 ± 2	24	23	26	24.3 ± 1.5
0.5	31	29	36	32 ± 3.6	29	31	32	30.6 ± 1.5
1.6	30	35	33	32.6 ± 2.5	27	31	31	29.6 ± 2.3
5	35	38	33	35.3 ± 2.5	26	30	26	27.3 ± 2.3
Positive Control	210	217	196	207.6 ± 10.6	210	215	240	221.6 ± 16

Negative Control: water—100 μL/plate; Positive Control: 9-aminoacridine HCl (50.0 μg/plate) in the absence of S9 and 2-amino anthracene (2 μg/plate) in the presence of S9. Historical negative in the absence of S9: Range 19–40 (mean ± s.d. = 32 ± 10). Historical negative in the presence of S9: Range 18–43 (mean ± s.d. = 31 ± 6). Historical positive in the absence of S9: Range 187–250 (mean ± s.d. = 210 ± 42). Historical positive in the presence of S9: Range 223–270 (mean ± s.d. = 216 ± 34).

**Table A15:** Number of revertants/plate for *E. coli WP2 Uvr A* strain treated with Limoncella Extract.

		−S9				+S9		
mg/plate	Replicate1	Replicate2	Replicate3	Mean ± sd	Replicate1	Replicate2	Replicate3	Mean ± sd
Negative control	36	38	38	37.3 ± 1.1	38	40	44	40.6 ± 3
0.0016	37	35	35	35.6 ± 1.1	43	47	43	44.3 ± 2.3
0.005	29	34	40	34.3 ± 5.5	51	41	43	45 ± 5.2
0.016	40	40	30	36.6 ± 5.7	48	52	50	50 ± 2
0.05	40	43	38	40.3 ± 2.5	53	48	51	50.6 ± 2.5
0.16	37	37	40	38 ± 1.7	47	51	43	47 ± 4
0.5	39	39	40	39.3 ± 0.5	47	47	47	47
1.6	35	39	50	41.3 ± 7.7	48	53	61	54 ± 6.5
5	42	43	43	42.6 ± 0.5	44	45	49	46 ± 2.6
Positive Control	180	179	204	187.6 ± 14.1	230	237	249	238.6 ± 9.6

Negative Control: water—100 μL/plate; Positive Control: 4-nitroquinoline-N-oxide (1.0 μg/plate) in the absence of S9 and 2 amino- anthracene (20 μg/plate) in the presence of S9. Historical negative in the absence of S9: Range 37–58 (mean ± s.d. = 45 ± 11). Historical negative in the presence of S9: Range 32–70 (mean ± s.d. = 44 ± 6). Historical positive in the absence of S9: Range 109–195 (mean ± s.d. = 177 ± 29). Historical positive in the presence of S9: Range 193–263 (mean ± s.d. = 210 ± 34).
